# Dysregulated left inferior parietal activity in schizophrenia and depression: functional connectivity and characterization

**DOI:** 10.3389/fnhum.2013.00268

**Published:** 2013-06-12

**Authors:** Veronika I. Müller, Edna C. Cieslik, Angela R. Laird, Peter T. Fox, Simon B. Eickhoff

**Affiliations:** ^1^Institute of Clinical Neuroscience and Medical Psychology, Heinrich Heine UniversityDüsseldorf, Germany; ^2^Department of Neuroscience and Medicine, Research Center Jülich, INM-1Jülich, Germany; ^3^Department of Psychiatry, Psychotherapy, and Psychosomatics, Medical School, RWTH Aachen UniversityAachen, Germany; ^4^Research Imaging Institute, University of Texas Health Science Center at San AntonioSan Antonio, TX, USA; ^5^Department of Physics, Florida International UniversityMiami, FL, USA; ^6^South Texas Veterans Administration Medical Center, San AntonioTX, USA

**Keywords:** functional connectivity, depression, schizophrenia, inferior parietal cortex, resting-state

## Abstract

The inferior parietal cortex (IPC) is a heterogeneous region that is known to be involved in a multitude of diverse different tasks and processes, though its contribution to these often-complex functions is yet poorly understood. In a previous study we demonstrated that patients with depression failed to deactivate the left IPC during processing of congruent audiovisual information. We now found the same dysregulation (same region and condition) in schizophrenia. By using task-independent (resting state) and task-dependent meta-analytic connectivity modeling (MACM) analyses we aimed at characterizing this particular region with regard to its connectivity and function. Across both approaches, results revealed functional connectivity of the left inferior parietal seed region with bilateral IPC, precuneus and posterior cingulate cortex (PrC/PCC), medial orbitofrontal cortex (mOFC), left middle frontal (MFG) as well as inferior frontal (IFG) gyrus. Network-level functional characterization further revealed that on the one hand, all interconnected regions are part of a network involved in memory processes. On the other hand, sub-networks are formed when emotion, language, social cognition and reasoning processes are required. Thus, the IPC-region that is dysregulated in both depression and schizophrenia is functionally connected to a network of regions which, depending on task demands may form sub-networks. These results therefore indicate that dysregulation of left IPC in depression and schizophrenia might not only be connected to deficits in audiovisual integration, but is possibly also associated to impaired memory and deficits in emotion processing in these patient groups.

## Introduction

Depression and schizophrenia are both associated with social and affective dysfunctions as well as deficits in emotional processing (Bach et al., 2009; Bourke et al., 2010; Kohler et al., 2010; Comparelli et al., 2013). Research on affective deficits in psychiatric populations to date, however, has mainly focused on unimodal emotion processing, while in daily life emotion perception is generally based on the multimodal evaluation of information, such as hearing a laugh and seeing a smiling face. Importantly, in this context, emotional information from different sensory channels can be either congruent or incongruent, leading to faster responses when processing emotional congruent compared to incongruent information (De Gelder and Vroomen, 2000; Dolan et al., 2001; Collignon et al., 2008). Clinical studies have shown that patients with schizophrenia show aberrant audiovisual integration (De Gelder et al., 2005; De Jong et al., 2009; Van Den Stock et al., 2011). In contrast, Müller et al. (2012) and Müller et al. (2013) could not find any significant group difference in the behavioral rating of emotional faces while distracted by congruent and incongruent sounds, neither in a group of patients with schizophrenia, nor in depression. Importantly, the neuronal correlates of crossmodal emotional processing in clinical populations are still sparse. In a recent study (Müller et al., 2013) investigating audiovisual (in) congruence processing in depression, we showed that compared to healthy controls, patients failed to deactivate the left inferior parietal cortex (IPC) and inferior frontal cortex when confronted with congruent happy audiovisual information, while there was no difference between groups for incongruent pairs. As we will show in this paper by using the same paradigm, a similar effect in the same region of the left IPC in patients with schizophrenia can be observed. In particular, schizophrenic patients also reveal decreased deactivation in congruent audiovisual conditions compared to controls. Thus, both schizophrenia and depression go along with IPC dysregulation during congruent audiovisual emotional processing, possibly indicating increased processing of unambiguous stimuli in these patient groups (Müller et al., 2013).

However, when interpreting these findings, one has to acknowledge that the IPC is a heterogeneous region, which is involved in a wide range of different functions ranging from language and memory to action planning, higher social-cognition and other integrative processes (Glover, 2004; Wagner et al., 2005; Daselaar et al., 2006; Buckner et al., 2008; Binder et al., 2009; Spreng et al., 2009; Caspers et al., 2010; Arsalidou and Taylor, 2011; Bzdok et al., 2012; Schilbach et al., 2012; Seghier, 2013). Hence, it has been suggested that the IPC can be subdivided in different sub regions. Based on cytoarchitectonic mapping, Caspers et al. (2006, 2008) divided the IPC into seven different sub-regions. With regard to these divisions, the area that we found to be dysregulated in depression as well as in schizophrenia strongly overlaps with area PGp. Based on its anatomical and functional connections with temporal and lateral occipital (Caspers et al., 2011) as well as with frontal and parahippocampal areas (Uddin et al., 2010) it has been argued that PGp may mainly be involved in auditory-sensory integration and memory processes. Given the size and potential heterogeneity of this region in the posterior IPC, however, it remains open as to how the specific location disturbed in depression and schizophrenia relates to these roles. Furthermore, as the function of a specific brain area depends on those regions it interacts with, its role should not only be assessed in isolation but also together with regions it stands in interplay with (Stephan, 2004; Seghier, 2013). Therefore, the present study aims to investigate connectivity and function of the particular region, which has been found to be dysregulated in cross-modal affective integration across two different clinical groups, i.e., depression and schizophrenia. In particular, task-dependent and task-independent functional connectivity as well as behavioral characterization of the region of interest was carried out in healthy subjects in order to gain better insight of the role of this region from a system perspective.

## Methods

### Volume of interest

The volume of interest used in the current study is based on two fMRI studies investigating the neural correlates of audiovisual incongruence processing in patients with depression as well as patients with schizophrenia. Before, describing the methods of the current study, we first describe the patient samples on which the VOI is based on as well as the experimental procedure of the audiovisual paradigm.

#### Audiovisual paradigm and fMRI analysis

The stimuli and procedure used in the fMRI studies is the same as previously described (Müller et al., 2011, 2013). Thirty different pictures of faces from five males and five females, each showing a happy, neutral, and fearful expression (FEBA; Gur et al., 2002) were combined with 30 auditory stimuli, consisting of 10 yawns, 10 laughs, and 10 screams. Additionally 10 different blurred faces served as masks. This resulted in 180 stimulus pairs with 9 different conditions (fearful/scream, fearful/yawn, fearful/laugh, neutral/scream, neutral/yawn, neutral/laugh, happy/scream, happy/yawn, happy/laugh). Every trial started with the presentation of a sound concurrently with a mask. After 1000 ms the mask was displaced by an either neutral or emotional face and presented with the continuing sound for another 500 ms. Subjects had to ignore the sound and to just rate the facial expression on an eight-point scale from extremely fearful to extremely happy.

fMRI data acquisition and statistical analysis was done as described in Müller et al. (2013). Images were acquired on a Siemens Trio 3T whole-body scanner (Erlangen, Germany) in the RWTH Aachen University hospital using blood-oxygen-level-dependent (BOLD) contrast [Gradient-echo EPI pulse sequence, *TR* = 2.2 s, in plane resolution = 3.1 × 3.1 mm, 36 axial slices (3.1 mm thickness)] covering the entire brain. Echo-planar imaging (EPI) images were corrected for head movement, normalized to the Montreal Neurological Institute (MNI) single subject template and spatially smoothed using an 8 mm FWHM Gaussian kernel. Data were then analysed using a General Linear Model as implemented in SPM8. For each subject, each experimental condition as well as the response event were separately modeled and simple main effects for each of the conditions computed by applying appropriate baseline contrasts. These individual first-level contrasts were then entered into a second-level group-analysis using an analysis of variance (ANOVA) employing a random-effects model. Based on these estimates of the second-level analysis, separate *t*-contrasts were calculated for the interaction congruence × group by applying the respective contrast to the 2nd level parameter estimates. The resulting SPM (T) maps were then thresholded at a cluster level FWE rate of *p* < 0.05 (cluster forming threshold: *p* < 0.001 at voxel level).

#### Subjects

The demographic and clinical characteristics of the patients with depression and the corresponding controls can be found in Müller et al. (2013).

Using the same audiovisual paradigm as in the previous study, we now tested a sample of 18 patients with schizophrenia and 18 healthy controls matched for age, gender, and education. Two patients and the corresponding controls were excluded from further analysis due to abnormal anatomy or an inability to understand the task. All participants were right handed, as confirmed by the Edinburgh Inventory (Oldfield, 1971) and reported normal or corrected-to-normal vision. Table 1 presents the clinical profile of the schizophrenic patient group. Patients were recruited from the inpatient and outpatient units of the Department of Psychiatry, Psychotherapy and Psychosomatics, RWTH University Hospital. Of the 16 patients included in the final analysis, 14 met ICD-10 criteria for paranoid schizophrenia (F 20.0), whereas two were diagnosed with the residual subtype (F 20.4). Only patients with no comorbid psychiatric or neurological illness and no substance addiction in the last 6 months were included in the study. All patients were medicated, in particular all of them were treated with atypical antipsychotics with one additionally taking typical antipsychotic medication. Furthermore, five patients were taking antidepressant agents and one was taking anticholinergic drugs.

**Table 1:** **Demographic and clinical characteristics of the patient and control groups**.

	**Patients**	**Controls**
Gender	6 female/ 10 male	6 female/ 10 male
Age	38.37 ± 10.21	37.94 ± 10.38
Education in years	11.75 ± 2.29	12.93 ± 2.20
Age of onset	29.19 ± 8.65	
Duration of illness	9.25 ± 6.59	
PANSS positive	10.63 ± 3.83	
PANSS negative	12.75 ± 5.59	
PANSS general	22.31 ± 6.33	

Healthy controls had no history of neurological or psychiatric disorder and did not take any mood- or cognition-altering medication.

All subjects gave informed consent into the study which was approved by the ethics committee of the School of Medicine of the RWTH Aachen University.

#### Definition of volume of interest

**Figure 1:**
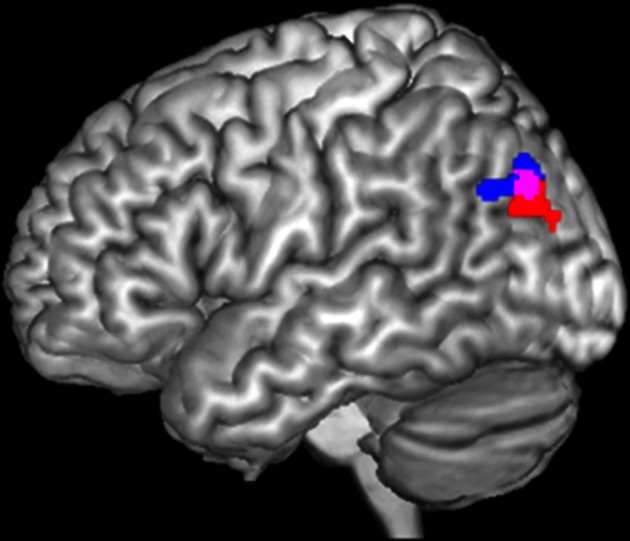
**Significant interaction between incongruence × group in left inferior parietal cortex in depression (blue) and schizophrenia (red).** The overlap of both activations served as seed area (purple) for functional connectivity calculation.

In the previous study in patients with depression (Müller et al., 2013), we found a significant incongruence × group interaction in the left IPC. In particular, patients failed to deactivate this region in the congruent happy audiovisual condition. Figure 1 (in blue) presents the dysregulated IPC region found in that study in depression (Müller et al., 2013). We now tested the same interaction in the schizophrenic sample, by using the same paradigm as well as applying the same methods and statistical analysis. When calculating the interaction contrast incongruence × group, a significant effect was again found in the left IPC (Figure 1, red). Similar as in depression, this interaction was driven by a failure to deactivate this region in congruent conditions.

The current study now aims to investigate functional connectivity and characterization of that particular area which has been found to be dysregulated in audiovisual congruence processing in both depression and schizophrenia. For that, a conjunction of the interaction contrast of both groups was performed. The resulting overlap then served as seed region (Figure 1, purple) for the calculation of functional connectivity.

### Task-dependent functional connectivity: meta-analytic connectivity modeling (MACM)

To characterize the co-activation profile of the left inferior parietal seed region (Figure 1, purple), we used meta-analytic connectivity modeling (MACM). This approach to functional connectivity assesses which brain regions are co-activated above chance with a particular seed region across a large number of functional neuroimaging experiments. MACM thus takes advantage of the fact that functional imaging studies are normally presented in a highly standardized format using ubiquitously employed standard coordinate systems, and the emergence of large-scale databases that store this information, such as BrainMap (Laird et al., 2009a, 2011) or Neurosynth (Yarkoni et al., 2011). The first step in a MACM analysis is to identify all experiments in a database that activate the seed region. Subsequently, quantitative meta-analysis is employed to test for convergence across the foci reported in these experiments. Significant convergence of reported foci in other brain regions therefore, indicates consistent co-activation, i.e., functional connectivity with the seed (Laird et al., 2009b; Eickhoff et al., 2010; Robinson et al., 2010). Thus, we first identified all experiments in the BrainMap database (www.brainmap.org) that featured at least one focus of activation in the seed region. Only studies reporting group analyses of functional mapping experiments of healthy subjects as well as activation only studies were included, while studies dealing with disease or drug effects were excluded. This resulted in inclusion of 160 experiments with a total of 2454 subjects and 2335 foci. Next, coordinate-based meta-analysis was performed in order to identify consistent co-activations across experiments by using the revised Activation Likelihood Estimation (ALE) algorithm (Eickhoff et al., 2009, 2012). This algorithm aims to identify areas showing a convergence of reported coordinates across experiments, which is higher than expected under a random spatial association. The results were thresholded at a cluster-level FWE corrected threshold of *p* < 0.05 (cluster-forming threshold at voxel-level *p* < 0.001).

### Task-independent functional connectivity: resting-state

Resting state images were obtained from the Nathan Kline Institute “Rockland” sample, which are available online as part of the International Neuroimaging Datasharing Initiative (http://fcon_1000.projects.nitrc.org/indi/pro/nki.html). In total, the processed sample consisted of 132 healthy subjects between 18 and 85 years (mean age: 42.3 ± 18.08 years; 78 male, 54 female) with 260 EPI images per subject. Images were acquired on a Siemens TrioTim 3T scanner using BOLD contrast [gradient-echo EPI pulse sequence, repetition time (*TR*) = 2.5 s, echo time (*TE*) =30 ms, flip angle = 80°, in-plane resolution = 3.0 × 3.0 mm, 38 axial slices (3.0 mm thickness), covering the entire brain].

Data was processed using SPM8 (Wellcome Trust Centre for Neuroimaging, London, http://www.fil.ion.ucl.ac.uk/spm/software/spm8/). Prior to further analyses, the first four scans were discarded and EPI images were then corrected for head movement by affine registration using a two-pass procedure in which in a first step, images were aligned to the initial volumes and then subsequently to the mean of all volumes. Next, for every subject, the mean EPI image was spatially normalized to the MNI single-subject template (Holmes et al., 1998) by using the “unified segmentation” approach (Ashburner and Friston, 2005). Ensuing deformation was then applied to the individual EPI volumes and images smoothed by a 5-mm full width at half maximum Gaussian kernel to improve signal-to-noise ratio and to compensate for residual anatomical variations. Time-series of each voxel were processed as follows (Reetz et al., 2012; Sommer et al., 2012; Zu Eulenburg et al., 2012): Spurious correlations were reduced by excluding variance, which can be explained by the following nuisance variables: (1) the six motion parameters derived from image realignment; (2) their first derivatives; (3) mean GM, WM, and CBF intensity. All nuisance variables entered the model as first and also as second order terms (cf. Satterthwaite et al., 2013 for an evaluation of this framework). Finally, data was band-pass filtered with the cut-off frequencies of 0.01 and 0.08 Hz. Just as for the MACM analysis, the seed region was provided by the conjunction of the incongruence × group interaction of the schizophrenia and depression study (Figure 1, purple). Time-courses of all voxels within that seed were then extracted and expressed as the first eigenvariate. To quantify resting-state functional connectivity linear (Pearson) correlation coefficients were computed between the ensuing characteristic time series of the seed region and the time-series of all other gray matter voxels of the brain. These voxel-wise correlation coefficients were then transformed into Fisher's *Z*-scores and then fed into a second-level ANOVA including an appropriate non-spericity correction as implemented in SPM8. Results were again thresholded at a cluster-level FWE corrected threshold of *p* < 0.05 (cluster-forming threshold at voxel-level *p* < 0.001). That is, the same criteria was used for both MACM and resting state analyses.

### Conjunction between MACM and resting-state

We here aimed to identify regions that show functional connectivity with the seed across different mental states. Therefore, a conjunction analysis between MACM and resting state results was performed in order to detect areas showing both, task-dependent and task-independent functional connectivity with the seed region (extent threshold of 20 voxels). That is, by using the minimum statistics (Nichols et al., 2005; Jakobs et al., 2012) and computing the intersection of the thresholded connectivity maps derived from two different concepts of functional connectivity, we aimed to delineate consistent functional connectivity of the seed.

Finally, all results were anatomically labeled by using the SPM Anatomy Toolbox (Eickhoff et al., 2005, 2006, 2007).

### Functional characterizations of derived co-activations

Based on the conjunction of MACM and Resting-State, ensuing regions showing consensus of functional connectivity with the seed were further investigated. In particular, we were mainly interested in the functional role of the left IPC in co-activation with its connected regions. That is, we assessed functional properties (Laird et al., 2009b; Cieslik et al., 2012; Rottschy et al., 2012) for the IPC activation combined with all other regions derived from the task-dependent and task-independent conjunction. Therefore, functional characterization of the derived network was performed by using the behavioral domain (BD) and paradigm class (PC) meta-data categories from the BrainMap database (Laird et al., 2009a, 2011; Turner and Laird, 2012), describing the classes of mental processes isolated by the archived experiments' statistical contrasts. BDs include the main categories cognition, action, perception, emotion, interoception as well as their subcategories. In contrast, PCs classify the specific task employed (see http://brainmap.org/scribe/ for the complete list of BD and PC). For the functional characterization of the different networks, we proceeded as follows: First, we identified all experiments in the BrainMap database, which featured at least one focus of activation within the IPC and its connected region(s). That is, we identified all experiments activating the left IPC and simultaneously those regions it is connected with. Forward inference and reverse inference were calculated for each network in order to characterize the functional profiles of the respective networks. While forward inference on the functional characterization is based on the probability of observing activity in a brain region (or network) given knowledge of a psychological process, reverse inference tests the probability of a psychological process being present given knowledge of activation in a particular brain region (or network). In particular in the forward inference approach, we determined a network's functional profile by identifying taxonomic labels for which the probability of finding activation in the respective network is significantly higher than the overall chance (across the entire database) of finding activation in that particular network. Significance was established using a binomial test (*p* < 0.001). In the reverse inference approach, a network's functional profile was determined by identifying the most likely BDs and paradigm classes given activation in a particular network. Significance was assessed by means of a chi-square test (*p* < 0.001).

## Results

### Task-dependent functional connectivity

**Figure 2:**
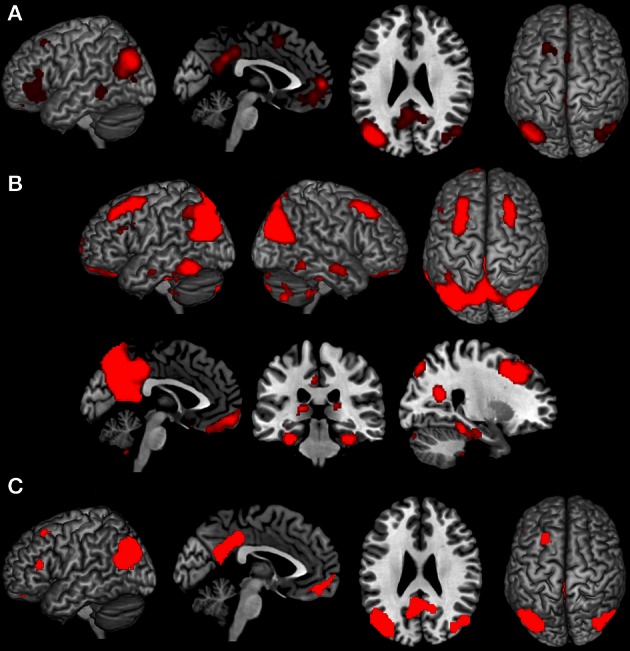
**Results of the task-dependent (A), task-independent (B) analysis as well as the conjunction across both approaches (C). (A)** Task-dependent functional connectivity of the seed region with bilateral parietal cortex extending into middle temporal and middle occipital gyri, with posterior cingulate cortex and precuneus, medial orbitofrontal cortex, left inferior frontal gyrus, left middle/superior frontal gyrus and left middle temporal gyrus. **(B)** Task-independent functional connectivity of the seed with bilateral parietal cortex extending into lateral occipital und temporal gyrus, precuneus and posterior cingulate cortex, as well as with bilateral cerebellum, hippocampus, parahippocampal gyrus, thalamus, fusiform gyrus, inferior and middle temporal gyrus, middle frontal gyrus, left inferior frontal gyrus, and medial orbitofrontal cortex. **(C)** Conjunction of task-dependent and task-independent connectivity reveals significant functional connectivity of the seed area with bilateral inferior parietal cortex, precuneus and posterior cingulate cortex, left middle and inferior frontal gyrus and medial orbitofrontal cortex.

Analysis of task-based functional connectivity by MACM revealed significant co-activation of left IPC with its surrounding parietal areas (overlapping PGp and PGa, Caspers et al., 2006, 2008) extending into middle temporal and middle occipital gyri, with its right homologue (overlapping PGp, Caspers et al., 2006, 2008) also extending into middle temporal and occipital gyri, as well as with precuneus and posterior cingulate cortex (PrC/PCC), medial orbitofrontal cortex (mOFC), left inferior frontal gyrus (IFG) (overlapping area 45, Amunts et al., 1999), left middle/superior frontal gyrus and left middle temporal gyrus (Figure 2A).

### Task-independent functional connectivity

Resting state connectivity of the seed region revealed significant connectivity with a broad network including bilateral inferior (overlapping PGp and PGa, Caspers et al., 2006, 2008) and superior parietal cortex (overlapping 7A, Scheperjans et al., 2008) extending into lateral occipital und temporal gyrus, Prc/PCC. Furthermore, functional connectivity was found with bilateral cerebellum (overlapping Lobule VI, IX, VIIa Crus I, Vlla Crus ll, Diedrichsen et al., 2009), hippocampus (overlapping CA, SUB, and EC, Amunts et al., 2005), parahippocampal gyrus, thalamus, fusiform gyrus, inferior and middle temporal gyrus, middle frontal gyrus (MFG), left IFG (overlapping area 44 and 45, Amunts et al., 1999), as well as mOFC (Figure 2B).

### Conjunction of task-dependent and task-independent functional connectivity

The main interest of the present study was to characterize functional connectivity of the seed region that can be observed in both, task-dependent (MACM) and task-free (resting state), connectivity approaches. Areas consistently observed in both functional connectivity analyses consisted of all areas found in MACM except left middle temporal gyrus. That is, convergence between resting state and MACM connectivity of our left parietal seed could be found with its surrounding parietal (overlapping PGp and PGa, Caspers et al., 2006, 2008), middle occipital and temporal regions, its right homologue (overlapping PGp and PGa, Caspers et al., 2006, 2008), the PrC/PCC, left MFG extending into superior frontal sulcus, as well as IFG (overlapping area 45, Amunts et al., 1999) and mOFC (Figure 2C).

### Functional characterization of derived networks

In a next step, forward and reverse inference were calculated for the whole network (IPC-PrC/PCC-mOFC-MFG-IFG) as well as each sub-network (left IPC co-activation with every combination of the regions of the derived network).

**Figure 3:**
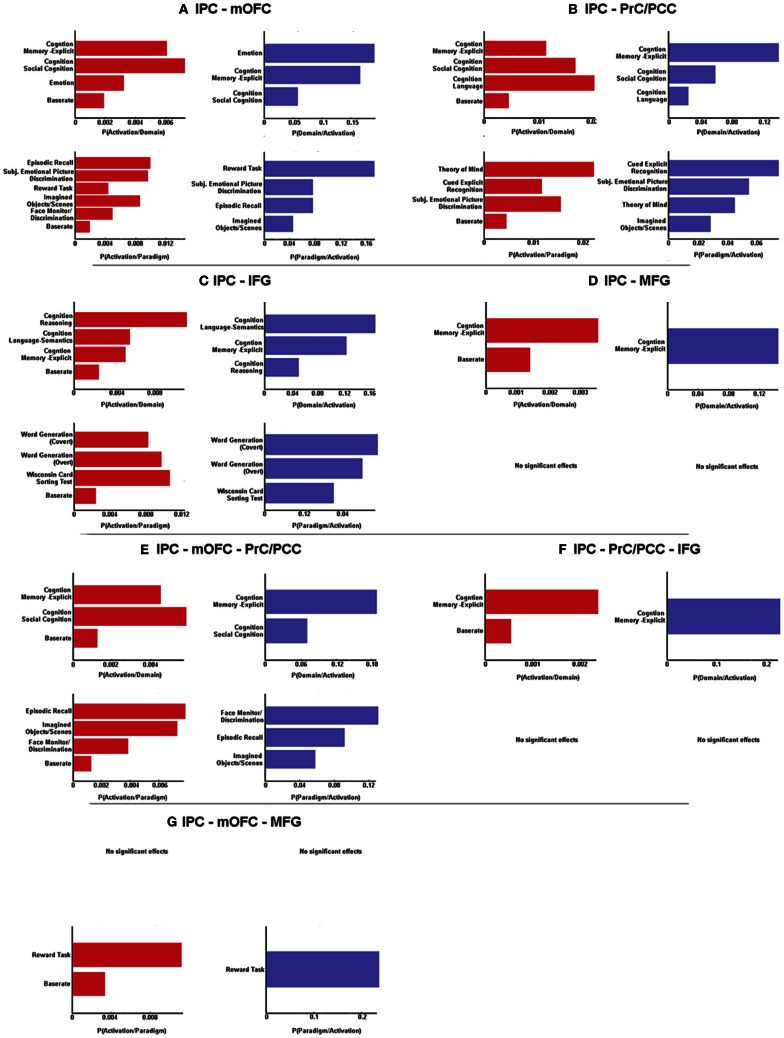
**Significant behavioral domains and paradigm classes for the different sub-networks revealed by forward (red) and reverse inference (blue).** Details can be found in the result section “Functional characterization of derived networks”.

BDs that were overrepresented among experiments co-activating with the left IPC and the mOFC were explicit memory, social cognition and emotion. Furthermore, co-activation of those two regions was associated with paradigm classes related to episodic recall, subjective emotional picture discrimination, reward, imagination of objects/scenes and face monitoring. The same BDs and paradigm classes were revealed by reverse inference, except face monitoring, which revealed significance for forward inference only (Figure 3A).

Analysis of BDs overrepresented among experiments that co-activate left IPC and the PrC/PCC area revealed significant meta-data labels related to cognition, in particular with its subcategories explicit memory, social cognition and language. Moreover, there was a significant association with paradigm classes referring to theory of mind, explicit recognition, and subjective emotional picture discrimination. Reverse inference again revealed the same BDs and paradigm classes, except with imagined objects/scenes representing an additional paradigm class (Figure 3B).

In contrast, significant BDs overrepresented among experiments that co-activate left IPC and left IFG were also related to cognition, but with the subcategories reasoning, semantic language processing as well as explicit memory. Paradigm classes significantly associated with co-activation of IPC and IFG were overt and covert word generation and the Wisconsin card sorting test (Figure 3C).

Analysis of IPC and left MFG revealed above chance co-activation of those region in experiments related to explicit memory for both forward and reverse inference, but no significant specific paradigm class (Figure 3D).

Co-activation of IPC, mOFC, and PrC/PCC featured a significant overrepresentation of experiments related to explicit memory and social cognition. Analysis of the paradigm classes further revealed association with tasks involving episodic recall, imagined objects/scenes and face monitoring (Figure 3E).

IPC, PrC/PCC, and IFG analysis revealed above chance co-activation of those three regions in experiments referring to explicit memory, but also no significant paradigm class (Figure 3F).

Additionally co-activation of IPC, mOFC, and MFG was only significantly associated with reward tasks, but with no specific BD (Figure 3G).

All other combinations (IPC-PrC/PCC-MFG, IPC-mOFC-IFG, IPC-MFG-IFG, IPC-PrC/PCC-mOFC-MFG, IPC-PrC/PCC-mOFC-IFG, IPC-PrC/PCC-MFG-IFG, IPC-mOFC-MFG-IFG and IPC-PrC/PCC-mOFC-MFG-IFG) didn't reveal any significant association to any BDs or paradigm classes.

## Discussion

The current study investigated the connectivity and functional properties of a region in the left IPC, which has been found to be dysregulated in schizophrenia and depression during processing of audiovisual emotional stimuli. That is, we investigated neuronal networks and their associated functions that center on an inferior parietal region showing aberrant responses during a multi-modal affective processing task in two major psychiatric disorders. First, results revealed that the seed region in the left posterior IPC functionally connects with PrC/PCC, mOFC as well as MFG and inferior (IFG) frontal gyrus. Quantitative functional characterization further indicates that on the one hand, all areas are engaged during experiments related to memory. On the other hand, sub-networks that relate to social cognition, reasoning, emotional, or language processing are also discernible.

### The role of the left IPC

The IPC is a large and heterogeneous region that has been found to be involved in a wide range of different processes, ranging from action, memory, and language to mathematical problem solving and social cognition. Furthermore, it has been described as part of the default mode network (Glover, 2004; Wagner et al., 2005; Daselaar et al., 2006; Buckner et al., 2008; Binder et al., 2009; Spreng et al., 2009; Caspers et al., 2010; Arsalidou and Taylor, 2011; Bzdok et al., 2012; Schilbach et al., 2012; Seghier, 2013). Apart from this functional diversity, the IPC is also heterogeneous with regard to its macroanatomy. It has for instance been classified into BA39, the angular gyrus and BA40, the supramarginal gyrus. In addition, based on cyto-architectonic mapping, Caspers et al. (2006, 2008) subdivided the IPC into seven different regions. Thus, both the functional as well as architectonical diversity strongly suggest that the IPC as a whole is too large and heterogeneous to allow any meaningful interpretations of IPC activity or IPC dysregulation.

Going more into detail, the IPC area which has been analyzed in the current study demonstrates correspondence with the angular gyrus and more precisely with area PGp (based on the subdivisions of Caspers et al., 2006, 2008). This part of the IPC is mainly involved in language processing (Hall et al., 2005; Binder et al., 2009; Price, 2010; Clos et al., 2012) and has in this context been suggested to act as a high-level supramodal conceptual integration area (Binder et al., 2009). In addition and in line with this view, investigation of audiovisual speech integration (Bernstein et al., 2008) point to angular gyrus involvement in crossmodal binding. Furthermore, Joassin et al. (2011) report greater left angular gyrus activity when presenting bimodal face–voice pairs compared to faces or voices alone, indicating a general and not only speech-specific role of this region in audiovisual binding. However, apart from crossmodal binding, the left angular region has also been associated with memory, the default mode network and social cognition (Seghier, 2013). Therefore, the common process involved in all these different tasks and domains associated with the angular gyrus may lie in the integration of information and concepts from different modalities and subsystems (Seghier, 2013). This indicates that it might be more informative to assess the functional role of the IPC from a network-based perspective. This idea is in line with the view that a specific role of a brain area cannot completely be determined by looking at it in isolation but should ideally be investigated together with regions it stands in interplay with (Stephan, 2004; Seghier, 2013). Therefore, it may be argued that, in order to be able to interpret activity in such a complex multimodal region as the IPC as well as its dysregulation in schizophrenia and depression, it is crucial to investigate its functional role from a system perspective.

### Connectivity of the left posterior IPC

By using two different approaches, investigation of functional connectivity revealed left IPC functional connectivity with numerous cortical and subcortical structures. In previous studies, area PGp, which overlaps with our seed, has been shown to structurally connect to temporal and lateral occipital areas (Caspers et al., 2011) but also with frontal (Caspers et al., 2011) and (para)hippocampal regions (Uddin et al., 2010). In addition, investing resting-state functional connectivity, Uddin et al. (2010) also investigated functional connectivity of area PGp, demonstrating on the one hand similar but also different connections as found in anatomical studies. With a few subtle differences, possibly due to a larger seed in the former study, the results of the task-independent functional connectivity of the present study greatly resembles the results of Uddin et al. (2010), indicating a consistent network across different samples. In addition, as Figure 2A shows, task based co-activation of area PGp demonstrated co-activation with regions which have also been found to be functionally connected in the task-independent analysis, except left middle temporal gyrus. Therefore, left IPC, PrC/PCC, mOFC, MFG, and IFG may be regarded as a core network, as convergent connectivity patterns across both task dependent and task-independent approaches could be demonstrated and therefore they reflect regions that show coupling across two fundamentally different mental states (Jakobs et al., 2012). Now the question arises if this core network as a whole is involved in certain functions or if only the interplay of the left posterior IPC with specific regions of this core network is associated with particular functions. Therefore, we further analyzed the derived network with regard to the functional characterization of the different sub-networks. In this context, sub-networks are defined as association of specific functions to co-activation of the left IPC not with the whole but rather with only some (or only one) regions of the network.

### Common and differential functional roles of derived networks

#### Common role: explicit memory

Functional investigation of the sub-networks derived from the functional connectivity analyses reveals that all regions are involved in explicit memory processing. That is, memory processing was found to be above chance associated with co-activation of IPC-PrC/PCC, IPC-mOFC, IPC-IFG, IPC-MFG but also with co-activation of IPC-PrC/PCC-mOFG and IPC-Prc/PCC-IFG. All regions of this core network have already been associated with memory (Wagner et al., 2005; Brand and Markowitsch, 2008; Spreng et al., 2009). However, functional characterization of co-activation of all five regions together didn't reveal any significant result. This indicates that there is not only one specific memory process in which the whole network is involved, but rather, different sub-networks are associated with differential memory processes. This may depend on the stimulus material used, for instance IPC-PrC/PCC-mOFC co-activation may play a role when social stimuli are processed, whereas conjoint activity of IPC, PrC/PCC, and IFG might be associated with recollection of verbal material. Furthermore, it should be noted that these results might also indicate that the label “explicit memory” in BrainMap is in some way too broad and therefore combines memory processes which are still heterogeneous.

In terms of processing of social stimuli it may be further speculated that co-activation of IPC and PrC/PCC mainly denotes recollection of the content of explicit memory. In contrast, IPC and mOFC interactions on the other side might be more involved in the emotional connotation of explicit memory. In line with this idea, Brand and Markowitsch (2006) suggested that OFC is always involved when a memory has a personal/emotional connotation. As the recall of explicit memory, especially autobiographical information, often involves both recollection of facts itself as well as the associated emotional hue, the result of elevated likelihood of IPC-mOFC-PrC/PCC activation during explicit memory experiments is not surprising.

In sum, current results suggest that even though the whole derived network is related to memory, different sub-networks are involved in differential memory processes.

#### Specific roles of sub-networks

Besides the common process of memory, functional characterization also revealed specific roles of the different sub-networks. First of all, our results reveal that emotional processing as well as reasoning is sub-network specific. That is, emotion was associated with co-activation of IPC and mOFC only, while reasoning was exclusively overrepresented among experiments co-activating IPC and IFG. Thus, this results extent previous reports of involvement of mOFC in emotional processing (Phan et al., 2002; Kellermann et al., 2012) and IFG in reasoning (Prado et al., 2011), by showing both regions to be associated to these processes in co-activation with the left IPC.

Furthermore, with regard to our investigated networks, language was found to be associated with IPC and IFG as well as with IPC and PrC/PCC, but not with IPC, PrC/PCC, and IFG co-activation. This result is hence reasonable, considering that IPC and IFG co-activation was mainly associated to the subcategory semantic language processing while IPC-PrC/PCC co-activation was not related to any specific subcategory. On the one hand, IPC and IFG have already been reported to play a major role in semantic processing and resting state functional connectivity between those two regions have been demonstrated to correlate with reading comprehension (Hampson et al., 2006). On the other hand, IPC and PrC/PCC are important nodes of the mentalizing system (Mar, 2011). Therefore, it may be argued that IPC and IFG co-activation is more involved in general language comprehension, whereas conjoint activation of IPC, PrC/PCC mainly denotes language processes requiring theory of mind. In line with this view, Mar (2011) report IPC and precuneus activity only during story-based and non-story based theory of mind tasks but not just for narrative comprehension.

Moreover, in line with studies reporting a role of IPC, PrC/PCC, and mOFC in mentalizing and self-referential processing (Ochsner et al., 2005; Mar, 2011; Bzdok et al., 2012), functional characterization further reveal that IPC-PrC/PCC and IPC-mOFC, but also co-activation of all three regions together are related to social cognition. Thus, the two sub-networks (IPC-PrC/PCC and IPC-mOFC) may differently contribute to the overall process of social cognition. That is, IPC-mOFC co-activation may subserve the affective component of social cognition whereas IPC-PrC/PCC is more associated with introspection and self/other referential processing. As social cognition usually involves both, emotional but also perspective taking, co-activation of all three regions may be necessary for this process.

In sum, given the suggested role of the left posterior IPC as a higher-level integration area (Binder et al., 2009; Seghier, 2013), it may be speculated that depending on the specific regions the IPC co-activates with, the aspects which have to be integrated change, leading to association of different functions with differential sub-networks. These results therefore further highlight the importance of network-based investigations, indicating that the functional role of a specific brain area is highly dependent on those regions it interacts with.

### Posterior inferior parietal dysfunction in psychiatric disorders

Structural and functional deficits of the IPC have already been demonstrated, in schizophrenia as well as in depression (Canli et al., 2004; Torrey, 2007; Wang et al., 2008; Palaniyappan and Liddle, 2012; Zeng et al., 2012). Furthermore, in terms of schizophrenia, dysfunctions of this region have been associated with symptoms of thought disorder and depersonalization (Torrey, 2007). We investigated audiovisual emotional integration in schizophrenia and depression and found dysregulation of left posterior IPC in both patient groups. From a regional perspective, given the role of the IPC in combining information from different subsystems and in cross-sensory binding (Joassin et al., 2011; Seghier, 2013), dysregulation of this region in schizophrenia and depression suggests a deficit in audiovisual integration in both patient groups. In particular, deactivation possibly in order to inhibit binding of acoustic information with congruent visual target information was found to be impaired in schizophrenia and in depression. As especially deactivation of the congruent condition is impaired it may be suggested that patients show increased binding of congruent information compared to controls. This increased binding might in some way have a positive effect, possibly leading to increased salience of congruent pairs and as a result to normal face processing strategies. This assumption fits well with a previous EEG study in schizophrenia (Müller et al., 2012), demonstrating similar P1 amplitudes between patients and controls in emotional congruent audiovisual conditions, whereas in incongruent conditions patients showed a reduced P1 response.

However, as the current study now demonstrates that the left IPC is functionally connected with frontal cortices and PrC/PCC, which depending on task demands, form sub-networks, it may be speculated that deficits in this area might not only affect audiovisual processing of emotions but rather be associated to diverse areas of functioning. In particular, even though the seed was defined by an area dysregulated specifically in an audiovisual task, this left posterior IPC deficit might rather reflect impaired integration in general. This might then further result in deficits also in those domains that are associated with the different sub-networks the IPC interacts with. That is, inferior parietal dysregulations might affect processing in the whole connected network and therefore also be associated with cognitive deficits and impairments in emotion processing. In line with this view, both disorders, schizophrenia as well as depression, go along with cognitive, social and emotional impairments (Bhalla et al., 2005; Lee et al., 2005; Bach et al., 2009; Bourke et al., 2010; Kohler et al., 2010; Wolkenstein et al., 2011; Young et al., 2011; Dimaggio et al., 2012; Fioravanti et al., 2012; Comparelli et al., 2013; Snyder, 2013), as well as with changes in connectivity within parts of the network (Karlsgodt et al., 2008; Zhou et al., 2010). In addition, illness severity in both, schizophrenia and depression correlates with the severity of impairments in cognition, social skills as well as emotional impairment (McDermott and Ebmeier, 2009; Gollan et al., 2010; Tanaka et al., 2012; Ventura et al., 2013). Interestingly, in schizophrenia associations of functions within these domains are more often found or larger for negative than for positive symptoms (Tanaka et al., 2012; Ventura et al., 2013). However, how these associations relate to impairments in posterior IPC and its related network remains an open question. Therefore, for future studies, it would be of interest to investigate the network derived in the current study by comparing functional connectivity between patients and healthy controls, as well as correlate functional connectivity measures between these nodes with neuropsychological scores and symptomology.

## Conclusion

In summary, the present results demonstrate functional connectivity of left IPC with PrC/PCC, mOFC, left IFG, and MFG across task-dependent and task-independent approaches, which, depending on task demands, form sub-networks. While the whole network is associated with memory processes, specific sub-networks are involved when social cognition, reasoning, as well as emotional and language processes are required. Results therefore indicate that dysregulation of left IPC in depression and schizophrenia might not only reflect deficits in audiovisual integration, but is possibly also connected to impaired emotional and cognitive processing in these patient groups. Thus, the current study highlights the fact that in order to gain a better understanding of a region (and the meaning of its dysregulation), it is important to investigate its functional role from a system rather than a regional perspective.

### Conflict of interest statement

The authors declare that the research was conducted in the absence of any commercial or financial relationships that could be construed as a potential conflict of interest.
